# On deducing causality in metabolic networks

**DOI:** 10.1186/1471-2105-9-S4-S8

**Published:** 2008-04-25

**Authors:** Chiara Bodei, Andrea Bracciali, Davide Chiarugi

**Affiliations:** 1 Dipartimento di Informatica, Università di Pisa, I-56127, Pisa, Italy; 2 Dipartimento di Scienze Matematiche e Informatiche, Università di Siena, I-53100, Siena, Italy

## Abstract

**Background:**

Metabolic networks present a complex interconnected structure, whose understanding is in general a non-trivial task. Several formal approaches have been developed to support the investigation of such networks. One of the relevant problems in this context is the comprehension of *causality dependencies* amongst the molecules involved in the metabolic process.

**Results:**

We apply techniques from formal methods and computational logic to develop an abstract qualitative model of metabolic networks in order to determine *possible* causal dependencies. Keeping in mind both expressiveness and ease of use, we aimed at providing: *i)* a minimal notation to represent causality in biochemical interactions, and *ii)* an automated tool allowing human experts to easily vary conditions of *in silico* experiments. We exploit a reading of chemical reactions in terms of logical implications: starting from a description of a metabolic network in terms of reaction rules and initial conditions, chains of reactions, causally depending one from the another, can be automatically deduced. Both the components of the initial state and the clauses ruling reactions can be easily varied and a new trial of the experiment started, according to a *what*-*if* investigation strategy. Our approach aims at exploiting computational logic as a formal modeling framework, amongst the several available, that is naturally close to human reasoning. It directly leads to executable implementations and may support, in perspective, various reasoning schemata. Indeed, our abstractions are supported by a computational counterpart, based on a Prolog implementation, which allows for a representation language closely correspondent to the adopted chemical abstract notation. The proposed approach has been validated by results regarding gene knock-out and essentiality for a model of the metabolic network of *Escherichia coli* K12, which show a relevant coherence with available *wet*-*lab* experimental data.

**Conclusions:**

Starting from the presented work, our goal is to provide an effective analysis toolkit, supported by an efficient full-fledged computational counterpart, with the aim of fruitfully driving *in vitro* experiments by effectively pruning non promising directions.

## Background

Understanding the relationships amongst the elements involved in biologic interaction networks, such as the functioning of cellular metabolism, is a relevant problem in Systems Biology. In the words of [1], “diagrams of interconnections represent a sort of static roadmaps, but what we really seek to know are the traffic patterns, why such patterns emerge, and how we can control them”. Having a formal description of the interconnections and a methodology to perform software simulation on how these patterns are, should help in orientating *in vitro* experimentation. Under this regard, *causality* can play an important role in finding chains of reactions that connect the parts of the system of interest, e.g. for determining causal correlations among molecules that are not apparently correlated. In general, the proposed models of complex systems code a lot of information and determining possible correlations and causal dependencies may be not straightforward or computationally expensive.

We focus here on metabolic networks, i.e. the set of the cellular biochemical pathways involved in energy management and in the synthesis of structural components. Biochemical pathways are typically composed of chains of enzymatically catalyzed chemical reactions and are interconnected in a complex way. Starting from the composition of local, well understood behaviour, the study of the overall emerging behaviour of metabolic networks appears difficult.

We apply techniques from formal methods and from computational logic in order to develop an abstract qualitative model of metabolic networks, focussing on causality. Keeping in mind both expressiveness and ease of use, we aimed at providing: *i)* a minimal notation to represent causality in biochemical interactions, and *ii)* an automated tool allowing the human expert to easily vary conditions of the *in silico* experiment. We introduce a simple and skeletal notation, inspired by biochemical reactions, to emphasize the causality aspects we are concerned with. The choice of relying on computational logic, which “provides a straightforward and intuitive representation of human reasoning” [2], has appeared particularly suitable in the multidisciplinary context of our work. On the one hand, it should be palatable to biologists, since very close to the biochemical intuition. On the other hand, it has a direct computational counterpart, which computer scientists can build upon in order to devise the needed analysis tool.

In our notation, biochemical reactions are given an abstract representation: we only record which are the relations between the source and the target elements of each single reaction, e.g. between two molecules *M* and *N* and the molecule *P* they produce. In turn, *P* can become a source molecule in another reaction and so on and so forth. In other words, we abstract away from quantities, stoichiometric proportions, kinetical or thermodynamics parameters that are involved in the production of *P*. Noticeably, we also abstract from the actual dynamics of reactions. Intuitively speaking, we project reactions on a “flat” temporal scale so that the availability of *M* and *N* is never spoiled after the production of *P*, and other metabolites can be caused by *M* and *N*. This is also an abstraction over quantities, since “infinite copies” of *M* and *N* result always available. Because of the abstraction, the reaction leading to *P* actually represents the *possibility* for *P* to be generated, or *caused*, *in vivo*, by the presence of *M* and *N*. Indeed, the model gives an over-approximation of the set of the actual pathways, possibly including some pathways that could be actually prevented, for instance, by the lack of a suitable quantity of reactants or by an inadequate temperature. The payoff of the abstraction adopted is in terms of expressiveness of the language and effectiveness of its computational counterpart. Abstracting from quantitative issues may prevent reasoning about some of the dynamical features of (bio)chemical objects. However, it makes possible to take into consideration some aspects of those systems (typically large biochemical networks) whose kinetical and thermodynamical parameters are scarcely known.

Chains of causal reactions can be, step by step, automatically deduced. To this aim, we exploit an analogy between logical implications and chemical reactions, by interpreting the reaction of *M* and *N* that produces *P* as a logical clause stating that *M* and *N* imply *P*. Our method is supported by a computational logic counterpart, based on a Prolog implementation of a bottom-up semantics. This allows us to compute the set of all the metabolites that can be produced as consequences of a given set of rules and starting from a set of initially available metabolites. This is step-wise determined by repeatedly adding the metabolites that can be immediately caused by the application of the rules to the set of the so-far produced metabolites. We then relate the computational construction with the original model and prove convergence properties of the process.

Despite the abstract working hypotheses adopted, our framework has revealed sufficient to provide meaningful approximations of the possible chains of reactions during experiments. Moreover, the approach facilitates thinking and revising the biological model itself, by making easy to vary both the components of the initial state and the clauses that rule reactions.

The proposed approach can serve as a sort of “what-if” analysis, repeatedly exploring different scenarios, each one derived from a different set of working hypotheses. Our tool allows us to rapidly evaluate the impact of changes in the hypotheses on a particular observable outcome. Thus, we obtain an interactive and effective analysis, that can be used to differentiate the most promising causal relations deduced, which deserve to be tested *in vitro*, from those that instead can be pruned.

We have validated our approach by studying the robustness of the metabolic network of *E. coli* K12. Selected genes have been knocked-out by disabling the rules regarding the corresponding encoded enzymes. Results are coherent with the actual biological behaviour, observed in *vitro* and reported in [3] and in the “Geno Base” (), a database entirely dedicated to *E. coli* K-12.

This paper is organized as follows. Next, we will discuss related work and then present our formal framework, illustrating how biochemical processes can be represented inside it. Results about experiments with the metabolic network of the *E. coli* K-12 metabolic genotype follow. Conclusions are followed by a section Methods, in which we report on the techniques used.

### Related work and comparison

Our work can be included in the recent research trend which exploits well established theories and techniques from formal methods in order to support the interpretation of the big amount of the raw biological data now available. By using logic, we slightly diverge from the line of research in which biological modelling is inspired by the use of concurrent models. Concurrency theory offers a wide choice of models naturally expressing causality, that is one of its essential notions. Nevertheless, causality is a natural notion also in the logic framework. Concurrent models are focussed on the description of the dynamic behaviour of whole systems. Considering these aspects can be computationally demanding. This has led us to the choice of an even more abstract model, where – as mentioned above – the notion of time is abstracted away. An abstraction similar to ours can be performed a posteriori on a concurrent model, for instance by resorting to static analysis techniques, which offer static approximations of the dynamic behaviour. Usually, static techniques extract information by systematically inspecting the specification of the dynamic behaviour of systems. In our approach instead, we want to infer information by directly inspecting the set of reactions, modulo the abstractions we discussed above, thus skipping the specification of the chemical dynamics. Resorting to static techniques represents a typical way to drastically reduce the computational cost due to the study of all the possible dynamic evolutions of a system. The price to pay amounts to a loss of precision, since they usually provide over-approximations of the behaviour.

Among the several formalisms developed in concurrency theory and applicable to Systems Biology, we recall below the most relevant for our purposes. Petri Nets [4] offer a way to graphically represent the structure of distributed systems. They have been successfully applied to the modelling of metabolic pathways and simple genetic networks (see e.g. [5,6] to cite only a few). They model pathways and networks in terms of their dynamic evolutions.

Process calculi describe interactions and communications between independent agents or processes. The underlying idea is that a biological system can be seen as concurrent systems. In particular, π–calculus [7] and Ambient Calculus [8] have been transferred from theoretical computer science setting to the biology setting, leading to suitably extended biological versions of them, such as the Biochemical stochastic π-calculus [9,10] and BioAmbients [11]. Also a version of CCS, RCCS [12] has been introduced to address biological issues. Other calculi have been instead specifically defined for biological modelling, such as κ-calculus [13], Brane calculi [14] and Beta Binders [15]. Chemistry has been already invoked explicitly in the process algebraic context many years before the coming of systems biology. In [16], an abstract machine based on the chemical metaphor is introduced: states are chemical solutions where floating molecules can interact according to reaction rules. Rules specify how to produce new molecules from old ones.

Closer to our approach is the work presented in [17], where the authors apply a causal semantics of the π-calculus in order to describe biochemical processes. The process computations that can be obtained quite accurately capture and reflect the behaviour of biological systems and causality has a key role in enhancing precision in such simulations. Our starting point is quite similar, but in our model the description of biological systems is given in terms of molecular entities and reaction rules that implicitly code the causal relationships, and hence the possible pathways. The causal semantics in [17] is based on an enhanced form of transition systems [18], that makes it possible to capture truly concurrent aspects like causality in an interleaving setting, like the process algebraic one. There are also other proposals, introduced with the same aim, see for instance the distributed transition systems in [19].

It is interesting to observe that our results could be comparable with the one obtained by using a quite efficient static technique like Control Flow Analysis (CFA) [20,21], applied to the π-calculus. In these settings, a reaction between *M* and *N* that produces *P* can be abstractly modelled as the synchronization of the process *M* and *N* on a shared channel *c*, with a process *P* as a continuation. Notice that in this case the full expressiveness of the π-calculus and, in particular, name-passing seems not to be needed. In its simplest form, CFA considers as effective all the communications that might occur through given shared channels, disregarding their actual viability, due for instance to synchronization dynamics. In modelling reactions in such a way, also CFA would not consider the possible consumption of reactants. Under this regard, our approach can offer an analogous result, within a more skeletal and abstract setting. Having an over-approximation of the *exact* behaviour of a system, both in the case of static analysis and in our framework, means that all those events that the prediction does not include will *never* happen, while when included, the events *can* happen, i.e. they are only possible.

Another recent proposal that shares some similarities with our approach is the one based on the biochemical abstract machine BIOCHAM [22], which also offers a formal modelling environment for biochemical processes, oriented towards qualitative aspects. It is based on a rule-centered language for specifying biochemical systems. Differently from our approach, BIOCHAM semantics takes into consideration the dynamics of systems and provides tools for querying temporal properties of these systems by using Computation Tree Logic. Refraining from dealing with quantities, BIOCHAM offers explicit controls on reactant consumption during reactions and, by default, all the possibilities are considered. This suggests further developments for our approach, where, currently, only the case of no consumption is admitted. The reason for this choice is effectiveness: in this way, branching semantics is avoided.

Still close to our approach and also to BIOCHAM is Pathway logic (see e.g. [23,24]), where rewriting logic is used for modelling biological processes. Rewrite rules describe local changes and the molecular patterns that cause them. Rules can be concurrently applied and this corresponds to the actual possibility of biological compartments to independently evolve. This offers a basis for *in silico* experiments and for advanced forms of symbolic analysis. We choose an alternative approach, by not resorting to a concurrent semantics, that is not in accordance with our aims.

Amongst the computational logic tradition, which largely influences our proposal, it is interesting to cite some recent proposals based on Abductive Logic Programming.

Complexity of bio-networks, understood as lack of complete knowledge, has been addressed by means of the capability of making assumptions provided by abduction. This approach has been applied to gene networks in [25], with motivations similar to ours. That proposal is based on a combination of Abduction and Induction: abduction allows inference from observable effects (see also [26]) and therefore it is used to generate hypotheses, while induction has the aim of learning general rules from these abduced hypotheses. The representation language has been ad-hoc devised. The predictive accuracy increases with the number of training examples. This methodology has a richer representation language than ours and aims at addressing a different class of problems in a different experimental setting. Differently, [2], and then [27], are based on a quite general class of languages, extended to deal with the biological context. These language, also known as action or event calculi, are suited to describe the non-monotone evolution of a dynamical environment, and specifically biological networks. Abduction is again used as an expressive means to compute/deduce explanations for missing information due to the dynamical nature of the world. Although perhaps more expressive than our approach, these proposals deal again with an explicit treatment of the dynamics of the systems, differently from our proposal that strives for simplicity in order to address, even approximately, causality in very large metabolic networks.

Finally, it is important to refer to the wide usage of graphs as representation language, traditionally close to biologist experience. Amongst the huge set of papers adopting graphs to model bio-networks, our work is closer to those that use arcs and nodes to directly represent reactions. For instance, an approach quite corresponding to our logical view of reactions – as far as the abstraction level is concerned – is in [28]. There, starting from a chosen qualitative interpretation of biochemistry analogous to ours, the authors focus on the topology of metabolic networks, aiming at defining a representative measure of network activity, the Synthetic Accessibility (the number of chemical reactions needed to transform a set of initial metabolites into a set of output ones). This notion has also been tested for predicting viability of mutant strains with accuracy results comparable to ours, although under a different perspective.

## Results and discussion

### A constructive formalisation of metabolic causality

We clarify our interpretation of the metabolic causal relations by introducing the notation used to represent biochemical reactions. We consider the assumptions on which such idealised notatation relies, discussing why, according to our aims, these assumptions can be considered viable working hypotheses. Such a notation represents our adopted formalisation of the biochemical reactions. Then, under the chosen hypotheses, we give a formal account of the fact that a metabolite, hereafter also called reactant, is *caused* by a network. In the section Methods, we will present the computational counterpart of the formalisation and we will relate the computational construction with the adopted model.

#### From biochemical reactions to causal relations

Let us consider a biochemical reaction written in the classical notation:

(1) mM+nN→pP+qQ

*M*, *N*, *P* and *Q* are the species involved and *m*, *n*, *p* and *q* the corresponding stoichiometric coefficients. Expression (1) indicates that, when the reaction occurs, a certain amount of *M* and *N* becomes a certain amount of *P* and *Q* according to the stoichiometric proportions. Besides stoichiometry, in order to exhaustively characterize a chemical reaction, one should take into account a number of factors related to thermodynamics and kinetics that represent the propensity for a reaction to occur and the rate at which reactants eventually become products.

Since we are interested in investigating causality relationships only, we can omit the description of many of the factors cited above. Then, we can give an abstract representation of (1) as follows:

(2) M∘N→P∘Q

We call such an expression a *reaction rule*. It simply states that the presence of both *M* and *N* represents the possibility for *P* and *Q* to be *produced* or *caused*. If applicable, i.e. it is known that the premises *M* and *N* are producible by a network, then also *P* and *Q* are producible. Informally speaking, a standard dynamical reading of (2) would be:

(3) M∘N→M∘N∘P∘Q

indeed, not considering the evolution of the network, reactants are not consumed at any step.

The description of causal relations within a metabolic network can be made by defining a set of metabolites initially present *I* and a set of reaction rules *R* that describe how new metabolites can be produced. Initial metabolites are represented in the form of rules with no premises like → *P*.

#### Example 1

Let us consider an experiment about a pathway occurring in an environment providing α-D Glucose, Glycerol and oxygen. By using suitable acronyms like *glc*, *gly* and *o*2, respectively, the initial conditions of such an experiment *I* will include:

→ *glc*

→ *gly*

→ *o*2

Moreover, let us imagine that the overall process to be described includes (some steps of) the upper part of the glycolytic pathway. Then, the set *R* will contain reaction rules like:

(4) glc6p→fru6p

(5) fru6p∘ATP→fru16p∘ADP

(6) fru16p→gap∘dhap

(7) dhap→gap

(8) gap→dhap

(9) gap∘nad→bpg13∘nadh

where the acronyms *glc*6*p*, *fru*6*p*, *fru*16*p*, *gap*, *dhap*, *nad*, *nadh*, *bpg*13 stand for Glucose 6-phosphate, Fructose 6-phosphate, Fructose1,6-bisphosphate, Glyceraldehyde 3-phosphate, Dihydroxyacetone phosphate, *NAD*^+^, *NADH* and 1,3 Bisphosphoglicerate, respectively.

The rules (4), (5), (6), (7) and (8) together, and (9) describe the reaction catalyzed by the enzymes phosphoglucose isomerase, 6-phosphofructo 1-chinase, fructose bisphosphate aldolase, triose phosphate isomerase, glyceraldehyde 3-phosphate dehydrogenase, respectively. Note that a reactant appearing on the right side of the operator → may well appear on the left side in another rule.

We eventually decompose the rules into simpler monadic rules, e.g. (2) will be written as:

(10) M∘N→P

(11) M∘N→Q

This transformation is causality preserving in the sense of the following proposition.

#### Proposition 1

Let *M*_1_ ○ … ○ *M_m_* → *P*_1_ ○ … ○ *P_n_* be an applicable rule, i.e. *M*_1_, …, *M_n_* are producible, and then *P*_1_, …, *P_n_* are producible. Then, also the simplified rules *M*_1_ ○ … ○ *M_m_* → *P_i_*, with *i* = 1, …, *n*, are applicable in any order, and their application makes *P*_1_, …, *P_n_* producible, as well.

The above proposition holds because causality behaves in a monotone way, since the application of subsequent rules cannot spoil the fact that any metabolite is producible.

The fact that a reactant is caused by a network is made precise by means of the following definition relating the metabolite to the chain of reactions that have made it exist, starting from an initial state *I* and according to a set of rules *R*.

#### Definition 1

Given a set of rules *R*, an initial state *I*, and a reactant *a* let us consider the following construction:

$$E_{R,I}\left(a\right)=\{\;\;\;\;\;\begin{array}{l} a\\ a\left[E_{R,I}\left(a_{1}\right),\ldots ,E_{R,I}\left(a_{n}\right)\right]\\ ⊥ \end{array}\;\;\;\;\begin{array}{l} \text{if}\to a\in I\\ \text{if}\;\;\;a_{1}\circ \ldots \circ a_{n}\to a\in R\;\;\text{and}\;\;E_{R,I}\left(a_{i}\right)\neq ⊥\;\;\forall i\in \left[1..n\right]\;\\ \text{otherwise} \end{array}$$

Then, any *E_R,I_*(*a*) ≠ ⊥ is an *explanation* for *a* under *R* and *I*.

The construction is non-deterministic (in the choice of the rule and in the choice amongst the first two cases), indeed there may be different ways to cause a metabolite.

#### False positives and false negatives: a problem of approximation

We here briefly discuss on the precision of our approach. As explained above, our approach is intended to offer an over-approximation of the dynamic behaviour of metabolic pathways. The existence of an explanation gives us indications about the *possibility* of the production of the metabolite, according to the many relaxing assumptions adopted. This means that we can have *false positives*, i.e. it is possible to predict *in silico* the production of metabolites that cannot be produced *in vivo*. This presence is expected as a consequence of abstraction and of over-approximation. However, the experimental results reported in the final part of the section give accuracy rates, i.e. a measure of approximation, comparable with those obtained by other well-known approaches in the literature.

On the contrary, up to the adequacy of the adopted biological model, we do not expect *false negatives*, i.e. metabolites that can be produced *in vivo* but have no explanation *in silico*, as stated by the following claim.

#### Claim

If there is no explanation for the production of a certain metabolite *a*, then *a* cannot be producible *in vivo*.

The intuition supporting our claim is based on the following reasoning: if *ad absurdum* the metabolite *a* is actually producible *in vivo*, then there is a certain set of biochemical reactions that can be applied starting from an initial set of metabolites. Starting from the same reactions and applying to them the abstractions illustrated above should lead us to the explanation we are looking for.

Note that experimental evidence of the possible production of a metabolite *in vivo* but not *in silico*, should suggest the need of a revision of the adopted biological model.

The set of reactants that can be “motivated”, in the sense above illustrated, by an explanation starting from an initial pool of given reactants and a specific set of rules can be automatically determined. Amongst the different and equivalent approaches that could have been used, we follow a logic-deductive interpretation, along the line of the explanatory approach adopted. For further details, see section Methods.

### Experiments

We have applied our approach to a biological model based upon the *E. coli* K-12 metabolic genotype proposed in [29] and [30]. This group of genes represents a subset of the whole genome of *E. coli* K-12 that includes genes encoding enzymes involved in energetic and biosynthetic metabolism. Using our formalism, we have represented the metabolic network composed by the enzymes encoded by the selected gene set and the metabolites involved in the catalyzed reactions. We have obtained a list of about 600 causal rules. We have performed some *in silico* “what-if” experiments and compared the obtained results with the correspondent *in vitro* counterpart, excerpts of which are reported in the following. The experiments have been carried out on a tool (see [31]), briefly presented in section Methods.

#### Mutually essential genes

In this *in silico* experimental session we performed a gene knock-out mimicking an homologous *in vitro* experiment presented in [3]. There, the authors silenced two target genes of *E. coli* K-12 (*sucAB* and *sucCD*) that encode for two enzymes involved in the Krebs cycle (α-ketoglutarate dehydrogenase and succinyl-CoA synthase, respectively). They found those genes “mutually essential” for the production of succinyl-CoA, i.e. *sucAB* and *sucCD* could be knocked-out individually, but not simultaneously in order to achieve Succinyl-CoA production. Succinyl-CoA is a critically important metabolite involved in several biochemical pathways leading, e.g., to energy production and peptidoglycan biosynthesis (via Diaminopimelate).

In order to simulate this gene knock-out, we have removed the rules corresponding to the reactions catalysed by α-ketoglutarate dehydrogenase and succinyl-CoA synthase. Then we have set the starting experimental conditions, including in the initial state all the metabolites that the cell is assumed to uptake from the external environment. Checking for the presence of succinyl-CoA at the end of the computation, we found that this metabolite was not produced (i.e. the correspondent fact was not deduced) only when both the target genes (i.e. the rules corresponding to the action of the encoded enzymes) were simultaneously turned off. This reflects what actually happens *in vitro*.

#### *In silico* gene knock-out

We have performed other *in silico* gene knock-outs and compared our results with the information contained in the “Geno Base” (), a database entirely dedicated to *E. coli* K-12. In this database genes are classified according to various criteria, among which their essentiality, i.e. their capability of causing cell death, when turned off. In our *in silico* knock-out experiments we tried to test gene essentiality verifying whether or not our *in silico* knock-out mutants exhibit features typically pertaining to living cells. We assumed that these characteristics should reasonably include the production of ATP (essential for cellular energetic metabolism), the production of reduced coenzymes NADPH and NADH and the production of not dispensable structural components, such as the cell wall (murein Biosynthesis). We have performed our *in silico* knock-out experiments over a sample of 132 genes of our set, each time removing the rules corresponding to the enzyme encoded by the silenced gene and checking for the presence of the observed elements at the end of each computation. We interpret the results as a prediction on the viability or not viability of the knock-out mutant under analysis. In our experimental setting (see Table 1), we slightly extend the notion of false positives and negatives introduced in the first part of the section and we say that a *True Positive* occurs when a knock-out mutant results viable both *in silico* and in the *in vivo* counterpart, while a *True Negative* occurs when a *in silico* knock-out mutant is not viable both *in silico* and *in vivo*, a *False Positive* occurs when a viable *in silico* knock-out mutant has an *in vivo* counterpart which is not, and a *False Negative* occurs when an *in silico* knock-out mutants is not viable, while its *in vivo* counterpart results viable. Note that this assignment has been arbitrarily chosen. To evaluate the performance of our method and compare it with similar approaches we use a performance measure taken by [28], that measures the number of true predictions – both positive (viability) and negative (non viability) – on the overall ones. Note that our definitions of false positives and false negatives differ from those proposed in [28]. This difference has been taken into account in calculating the *accuracy A* defined below.

**Table 1:** True and false positives and negatives according to our definition, depending on the viability of knock-out mutants in silico and in vivo

*CASE*	*IN SILICO*	*IN VIVO*
*True Positive*	*viable*	*viable*
*True Negative*	*not viable*	*not viable*
*False Positive*	*viable*	*not viable*
*False Negative*	*not viable*	*viable*

#### Definition 4

Let *TP*, *TN*, *FP*, *FN* be the number of true positives, true negatives false positives, and false negatives, respectively. We define *accuracy A* as

A=(TP+TN)/(TP+TN+FP+FN).

Our experiments give the following results:

$$\{\;\;\;\;\;\begin{array}{lll} TP & = & 102\\ TN & = & 13\\ FP & = & 17\\ FN & = & 0 \end{array}$$

As reported in [28], the accuracy of the Synthetic Accessibility approach therein presented ranges from 60% to 74%, while another approach for metabolic networks, i.e. the Flux Balance Analysis [30], has an accuracy that ranges between 62% and 86%. We found 17 false positives, resulting in an accuracy of about 87% over this specific experiment. These differences are probably strongly influenced by the way data sets are interpreted and by which cases are included in the data sets. However, the obtained results make us confident that our approach is reasonably accurate.

To give an intuitive idea of the experiments, we just report in Table 2, some examples of true positive, true negative and false positive cases (reminding that we have no false negatives). The symbols + or − stand for the presence or absence, respectively, of the observed elements in the *in silico* results.

**Table 2:** Gene knock-out experimental results

*Gene*	*ATP*	*NADPH*	*NADH*	*cellwall*	*outcome*
*acpS*	−	−	−	−	*true negative*
*lpxA*	+	+	+	−	*true negative*
*glk*	+	+	+	+	*true positive*
*aceA*	+	+	+	+	*true positive*
*prsA*	+	+	+	+	*false positive*
*pfk*	+	+	+	+	*false positive*

The presence of false positives (*in silico*, the mutant is predicted viable, but actually, *in vivo*, it is not) is expected in our framework, as a consequence of the abstraction and of the over-approximation we used in our model. This corresponds to the fact that something that has influence on viability and that is potentially producible *in silico*, it is not actually produced in real life. Finally, note that, in the experiments carried out, we have found no false negatives. This makes us confident of the correctness of the adopted biological model.

## Conclusions

In metabolic networks, metabolites are produced from a set of initial metabolites, through a set of chemical reactions. These reactions produce intermediate metabolites that can be both products or reactants. We have introduced a simple and skeletal notation to describe these networks in terms of molecular entities and reaction rules that specify their interactions and that implicitly code possible causal dependencies amongst reactions. This notation permits us, on the one hand, to give to chemical reactions an abstract and intuitive representation where quantitative aspects are abstracted away; on the other hand, this representation can be straightforwardly translated into an input for the tool we developed, paving the way for *in silico* experiments and further tool development. To this aim, we have exploited the analogy between reaction rules and logical implications, that allows us to automatically deduce chains of causally related reactions by means of a logical-based tool. Even though we do not consider the dynamic evolution of metabolic networks, our model is sufficient to give information on which metabolites can be possibly produced and how and, therefore, to give hints on the possible flows of reactions.

Moreover, our methodology makes it possible to reason about the model itself, by allowing us to vary both the initial conditions and the rules. It is easy to program such modifications and evaluating the impact of changes in the hypotheses is quite immediate, because the tool quickly reacts to the queries (typically an answer about a reasonable large network returns almost istantanelously). The what-if approach satisfies the need to simulate and investigate the behaviour of not fully known metabolic networks under different working hypotheses. In particular it allows us to perform perturbative experiments, whose results are not trivial to predict. In fact, if the studied network is complex enough, it results unfeasible to *a priori* estimate the effects produced by a local perturbation on the overall network. Finally, we have applied our methodology to the metabolic network of the *E. coli* K-12 metabolic genotype. The *in silico* experiments presented reflect the *in vitro* ones. The results obtained up to now show our method not to underperform analogous ones. Noticeably it is ground on a formalism that provides efficient and straightforward implementations.

Our ultimate goal for further investigations is that of supporting a heuristic process of searching causal explanations of metabolic phenomena, with in mind the “emphasis on hypothesis-driven research in biology” advocated in [1].

## Methods

To illustrate our methodology, we describe the computational framework that represents the counterpart of the formalisation of metabolic causality, introduced in section Results and Discussion. Finally, we relate the computational construction with the adopted formalisation.

The set of reactants that can be caused – according to our formalisation – by an explanation starting from an initial set of reactants and a set of rules can be automatically determined. Amongst the different and equivalent approaches that we could choose, we eventually follow a logic-deductive interpretation, along the line of the explanatory approach adopted. Technically, what follows consists of a fragment of Horn-based Logic Programming (having only a finite set of ground predicates) equipped with bottom-up semantics (see, e.g. [32]). However, for the sake of accessibility, the theory is recast in terms of rules (i.e., clauses) and reactants (i.e., predicates). In the following, reactants are directly represented, while causal rules of the model, *a* ○ *c* → *b* say, are straightforwardly translated into Horn-rules, like *a*, *c* → *b*, according to the following definition.

### Definition 2

Let *A* a finite set of *reactants* such that *a*_1_,…, *a_n_*, *a* ∈ *A*, then

$$a_{1},\ldots ,a_{n}\to a$$

with *n* ≥ 0 is a *rule*.

Notice that we can have rules with empty premises. These rules are used for representing the elements present in the initial state *I*.

The set of consequences of a given set of rules (respectively, the semantics of a logic program) can be defined according to a step-wise bottom-up process. The application of a rule to a set of reactants causes a new reactant when all the premises of the rule can be verified in the set. Starting from the set of the initially available reactants, the set of all the reactants that can be caused can be obtained by repeatedly adding the reactants that can be immediately caused by the application of all the rules.

### Definition 3

Let *R* be a set of rules and *A* a set of reactants, then the *immediate consequence operator T_R_*(*A*) : 2^*A*^ → 2^*A*^ is defined as

$$T_{R}\left(A\right)≜\left\{a|a_{1},\ldots ,a_{n}\to a\in R\;\;\text{and }a_{1},\ldots ,a_{n}\in A\right\}\cup A$$.

Moreover, $T_{R}^{n}\left(A\right)≜T_{R}\;\left(T_{R}^{n-1}\;\left(A\right)\right)\;\;\text{and}\;T_{R}^{o}\;\left(A\right)\;\;≜\;\;A$.

The convergence of the outlined process is guaranteed by the following result, which is based on the observation that, trivially, $\langle 2^{A},\;\subseteq \rangle$ is a (finite) *complete partial order(c.p.o.)* and *T*(_) is continuous over it (keeping the original *A* guarantees monotonicity). Moreover, being the set of reactants finite – i.e. the number of all the reactants occurring in rules *R* and in the initial state *I* – the bottom-up construction is also guaranteed to converge in a finite number of steps (then the fix point is indicated as $T_{R}^{n}$(*A*) instead of as $T_{R}^{\infty }$(*A*)).

### Proposition 2

Let *R* be a set of rules, *A* a set of reactants, and *m* the number of all the reactants in the model under consideration. Then, *n* <*m* exists such that

$$T_{R}\left(T_{R}^{n}\left(A\right)\right)=T_{R}^{n}\left(A\right)$$.

### Example 2

Let us consider a network with reactants *I* = { → *b*} initially available and consisting of the following simple rules *R*:

(12) a,b→c

(13) b→a

(14) a→b

(15) c→d

Applying *T_R_* ({*b*}) we have

$$\begin{array}{rrr} T_{R}\left(\left\{b\right\}\right) & = & \left\{a,b\right\}\\ T_{R}^{2}\left(\left\{b\right\}\right) & = & \left\{c,a,b\right\}\\ T_{R}^{3}\left(\left\{b\right\}\right) & = & \left\{d,c,b,a\right\}\\ T_{R}^{4}\left(\left\{b\right\}\right) & = & T_{R}^{3}\left(\left\{b\right\}\right) \end{array}$$

### Coherence of computational and metabolic causality

As standard, one would like to relate the computational construction provided with the original model, i.e. our adopted formalisation of biochemical reactions, introduced in section Results and Discussion. Let us start to observe that keeping track of the rule used when applying *T_R_*(_) would allow us to reconstruct explanations (which indeed recall SLD-trees of the top-down semantics of Logic Programming). Considering again the example above, the reactant *c* is caused in $T_{R}^{3}\left(\left\{b\right\}\right)$ by the application of rule (12), that in turn depends on rule (13), for the production of *a* that does not belong to the initial set *I* = {→ *b*}. Consequently, one would have the explanation *c*[*a*[*b*],*b*] for *c*. However, it is important to have considered convergence over reactants rather than over explanations. It is easy to observe that at the fourth step we would have the explanation *a*[*b*[*a*[*b*]]] due to the cycle between *a* and *b*. This amounts to say that – in the presence of cycles – explanations can growth indefinitely, i.e. convergence occurs at infinity. Hence, we have restricted ourselves to compute the reactants that can be produced, which is a finite process. However, the following correspondence between computational results and the potentially infinite model of explanations can be drawn.

### Theorem 1

Let *R* be a set of rules, *a* a reactant, *I* a set of reactants and $\bar{n}$ the minimum natural number such that $T\left(T_{R}^{\bar{n}}\left(I\right)\right)=T_{R}^{\bar{n}}\left(I\right)$. Then the following holds

$$a\in T_{R}^{\bar{n}}\left(I\right)\Leftrightarrow \exists E_{I,R}\left(a\right)\neq ⊥$$

### Proof

(Outline).

(⇒) By induction on the number of steps needed to firstly cause *a* in the bottom-up process (the rule that has motivated the inclusion of *a* can also be used as the top-most rule in the definition of an explanation for *a*. All the reactants in the premises of the rule must have been caused in less steps, and then, by inductive hypothesis, explanations for them exist, and these can be used to construct the explanation for *a*).

(⇐) By induction on structural complexity of *E_I,R_*(*a*). (By inductive hypothesis, all the reactants in the premises of the top-most rule in the explanation belong to $T_{R}^{\bar{n}}\left(I\right)$, then either *a* is in $T_{R}^{\bar{n}}\left(I\right)$ or $T_{R}^{\bar{n}}\left(I\right)$ is not a fix point, since the rule is clearly applicable and would cause *a*).

To address the problem of causality in metabolic networks, as seen above, we developed a software tool based on an implementation of a standard bottom-up semantics, running on top of SICTUS Prolog Interpreter (see [31]).

## Competing interests

The authors declare that they have no competing interests.

## Authors' contributions

All the authors equally contributed to the present work and wrote the paper. More precisely, CB and AB taken care of the computational framework and DC carried out the *in silico* experiments, while they jointly develop the formalisation of the metabolic causality and the analysis of the experimental results.
